# Machine learning-based prediction model for recurrence after radiofrequency catheter ablation in patients with atrial fibrillation

**DOI:** 10.3389/fcvm.2025.1642409

**Published:** 2025-08-08

**Authors:** Lujing Nie, Tianwei Zhang, Wenhua Wang, Xuefu Han, Meng Liu, Shujie Zhang, Wenjiu Feng, Yujie Wang, Yanbo Chen

**Affiliations:** ^1^ Department of Arrhythmia, Weifang People's Hospital, Shandong Second Medical University, Weifang, Shandong, China; ^2^ Department of Urology, The Affiliated Hospital of Qingdao University, Qingdao, Shandong, China; ^3^ Department of Cardiology I, Weifang People's Hospital, Shandong Second Medical University, Weifang, Shandong, China; ^4^ Department of Cardiology, Changle People's Hospital, Weifang, Shandong, China

**Keywords:** atrial fibrillation, machine learning, radiofrequency ablation, recurrence, prediction model

## Abstract

**Background:**

This study seeks to develop and validate a machine learning (ML) model for predicting atrial fibrillation (AF) recurrence at 12 months following radiofrequency catheter ablation (RFCA).

**Methods:**

A total of 430 consecutive patients with atrial fibrillation undergoing first-time radiofrequency catheter ablation were retrospectively enrolled between June 2022 and December 2023. Patients were randomly assigned to either a training cohort (70%) or a testing cohort (30%). Four ML algorithms were employed to develop prediction models. Model performance was evaluated using the area under the receiver operating characteristic curve (AUC) and accuracy. The SHapley Additive exPlanations (SHAP) methodology was employed to interpret the best-performing model and quantify each feature's contribution to its predictions.

**Results:**

Among the four machine learning algorithms evaluated, the Light Gradient Boosting Machine (LightGBM) model showed promising predictive performance on the testing set, achieving an accuracy of 0.721 and an AUC of 0.848 (95% CI: 0.778–0.919). Interpretation of the LightGBM model using SHAP analysis identified B-type natriuretic peptide (BNP) and the neutrophil-to-lymphocyte ratio (NLR) as the most impactful predictors for AF recurrence. The analysis revealed that higher levels of BNP and NLR were strongly associated with an increased risk of recurrence, whereas higher levels of albumin and lymphocyte count were protective. Other significant predictors included left atrial diameter (LAD) and nonparoxysmal atrial fibrillation (NPAF).

**Conclusion:**

Machine learning-based models show modest but promising performance for assessing AF recurrence risk after RFCA using routine clinical data. While requiring extensive external validation before clinical application, these models highlight the potential of ML to inform future risk stratification and guide personalized follow-up strategies.

## Introduction

Atrial fibrillation (AF) remains the most prevalent sustained arrhythmia, with globally increasing incidence and prevalence, resulting in a substantial public health and economic burden (1). The most serious complications associated with AF are stroke and heart failure (2). Despite catheter ablation's status as a Class 1 first-line therapy for AF rhythm control in select patients (3), post-procedural recurrence rates remain substantial (30%–40%) (4). Therefore, the accurate identification of patients at elevated risk for AF recurrence is crucial for selecting appropriate ablation candidates, managing postoperative expectations, and tailoring individualized follow-up care strategies.

Machine learning algorithms are being increasingly employed in medicine for diagnosis, treatment, and automated classification, facilitated by advances in statistical theory and computer technology (5). Research has established the predictive potential of artificial intelligence and machine learning for catheter ablation outcomes. Saiz-Vivo et al. (6) applied ML to heart rate variability data from implantable monitors to predict post-ablation AF recurrence. Hwang et al. (7) integrated speckle-tracking echocardiography with deep learning to identify imaging features predictive of post-ablation AF recurrence. Liu et al. (8) developed an ML-based model using CT scans to detect non-pulmonary vein AF triggers and predict post-ablation arrhythmia recurrence. Although their machine learning-based prediction model demonstrated good predictive performance, most studies focused on relatively expensive or complex examinations, which limits its generalizability to broader populations. In recent years, several composite inflammatory markers (e.g., NLR, SII, MHR) have demonstrated promising diagnostic value in predicting atrial fibrillation recurrence after catheter ablation, with the added advantage of being readily obtainable. Therefore, our objective was to develop and validate a predictive model for AF recurrence after RFCA using multiple ML algorithms. This model incorporates demographic characteristics, imaging data, laboratory measurements, and selected novel inflammatory biomarkers. We systematically compared the performance of each ML approach and identified the optimal predictive model.

## Methods

### Patient selection and data collection

**Figure 1:**
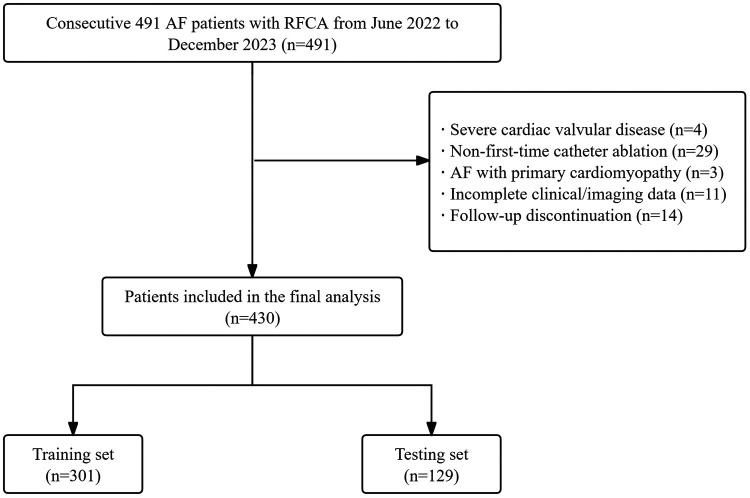
Flow diagram of patient's selection. AF, atrial fibrillation; RFCA, radiofrequency catheter ablation.

This retrospective study included 430 consecutive patients undergoing first-time RFCA for non-valvular AF at Weifang People's Hospital between June 2022 and December 2023. The inclusion criteria comprised patients aged 18 years or older undergoing first-time RFCA for non-valvular AF. Exclusion criteria comprised: (1) Severe valvular heart disease; (2) Non-first-time catheter ablation; (3) AF with primary cardiomyopathy; (4) incomplete clinical/imaging data; and (5) follow-up discontinuation. The patient selection flow diagram is depicted in Figure 1.

Data were extracted from the hospital's electronic medical record (EMR) system. All data underwent a manual quality review by two investigators to correct implausible values and data entry errors. Missing data for key variables were addressed through telephone follow-up, which successfully completed the datasets for 18 patients. The remaining 11 patients with irrecoverable key data were excluded. Consequently, all subsequent analyses were performed using a complete case analysis (CCA) approach.

This study received approval from the Medical Ethics Committee of Weifang People's Hospital (approval number KYLL20241008-13) and was conducted in accordance with the Declaration of Helsinki of the World Medical Association.

All patients’ baseline data were collected from their electronic medical records, including sex, age, body mass index (BMI), smoking and drinking history, sleep disorders, diabetes mellitus (DM), hypertension, coronary heart disease (CHD), chronic kidney disease (CKD), stroke/transient ischemic attack (TIA) history, heart failure, and AF classification (paroxysmal/persistent), white cells, lymphocytes, monocytes, neutrophils, platelets, hemoglobin, red cell distribution width-coefficient of variation (RDW-CV), uric acid, blood urea nitrogen, creatinine, fasting blood glucose (FBG), lipid profile (triglycerides, Low density lipoprotein cholesterol, high-density lipoprotein cholesterol), gamma-glutamyl transferase (GGT), BNP, albumin, free triiodothyronine (FT_3_), free tetraiodothyronine (FT_4_), and thyroid-stimulating hormone (TSH), LAD, left ventricular end-diastolic diameter (LVEDD), left ventricular ejection fraction (LVEF), estimated glomerular filtration rate (eGFR), neutrophil-to-lymphocyte ratio (NLR), platelet to lymphocyte ratio (PLR), monocyte-to- high-density lipoprotein ratio (MHR), systemic immune-inflammation (SII, neutrophil count × platelet count/lymphocyte count). Furthermore, patient-specific CHA₂DS₂-VASc and APPLE scores were calculated per established criteria. The CHA₂DS₂-VASc score incorporated: hypertension, heart failure, diabetes, vascular disease, age 65–74 years, and female sex (1 point each); prior stroke/TIA or age ≥75 years (2 points each) (9). The APPLE score included: age ≥65 years, persistent AF, left atrial diameter ≥43 mm, eGFR ≤ 60 ml/min/1.73 m^2^, and EF <50% (1 point each) (10).

### Post-procedural management and follow-up

Following RFCA, all patients adhered to a standardized management protocol. This included a 3-month course of an antiarrhythmic drug (amiodarone) and a direct oral anticoagulant (rivaroxaban), unless specific contraindications were present. Postdischarge follow-up was conducted at 1, 3, 6, and 12 months, involving clinical evaluation, 12-lead ECG, and 24-hour Holter monitoring. Symptomatic AF patients received additional outpatient assessments. If the patients had any AF-related symptoms, we performed further ECGs and Holter ECG examinations. Atrial arrhythmias (AF, atrial flutter, or atrial tachycardia) during the 90-day post-ablation blanking period were excluded from recurrence analysis.

### Feature selection

Initial statistical analyses included *t*-tests, Mann–Whitney *U* tests, and chi-square tests to evaluate intergroup differences in clinical characteristics. In our analysis, statistical significance threshold was set at two-tailed *p* < 0.05. Spearman correlation analysis was used to mitigate feature collinearity. To select the most salient predictors from our initial set of candidate variables, we employed the Least Absolute Shrinkage and Selection Operator (LASSO) regression model. A critical prerequisite for LASSO is the standardization of input features to prevent bias from variables with different scales. Therefore, prior to the LASSO analysis, all continuous variables were standardized using the *Z*-score method. This transformation rescaled each feature to have a mean of zero and a standard deviation of one. We chose LASSO not only for its predictive accuracy but also for its ability to produce a parsimonious and interpretable model, which is highly desirable for clinical application. This method performs L1 regularization, shrinking the coefficients of irrelevant features to zero and thus selecting a smaller, more robust subset of predictors. This approach also effectively manages multicollinearity by selecting a single representative from groups of correlated clinical variables.

### Model building

The entire cohort was randomly divided into a training set (70% of patients) and a testing set (30% of patients). To ensure the representativeness of both sets, a stratified sampling technique was employed based on the primary outcome (recurrence vs. no recurrence), thereby maintaining the same class distribution in both the training and testing sets as in the original dataset.

Given the class imbalance observed in our data (23% recurrence rate), we applied the Synthetic Minority Over-sampling Technique (SMOTE) to the training data before model fitting. This technique synthesizes new instances for the minority class to create a balanced training set, which helps prevent the model from being biased towards the majority class. Importantly, the test set was not oversampled and retained its original class distribution to serve as an unbiased benchmark for evaluating the model's true predictive performance on real-world data.

To predict AF recurrence following RFCA, we developed and compared four distinct machine learning algorithms known for their robust performance: support vector machine (SVM), light gradient boosting machine (LightGBM), GradientBoosting, and Adaptive Boosting (AdaBoost). The algorithms encompass various modeling methodologies, enabling detection of intricate data relationships and improved predictive performance. The SVM algorithm exhibits robust classification capabilities, particularly with high-dimensional datasets and limited samples. LightGBM, AdaBoost, and Gradient Boosting are all ensemble learning methods. While both LightGBM and Gradient Boosting employ decision trees as their base learners, AdaBoost demonstrates greater flexibility by accommodating various types of weak classifiers. These methods exhibit strong predictive performance when applied to appropriately structured datasets with sufficient sample sizes, with LightGBM being particularly distinguished by its computational efficiency. The selection of these algorithms enables a comprehensive evaluation of different models’ performance in predicting atrial fibrillation recurrence after RFCA, thereby ensuring optimal predictive outcomes. To ensure a robust and unbiased evaluation of our models, we employed a stratified five-fold cross-validation strategy. This procedure was implemented on the training dataset. Specifically, the data was partitioned into five subsets, or “folds”, of equal size. The “stratified” nature of this process guarantees that the distribution of the outcome classes within each fold is preserved to reflect the class distribution of the overall training dataset. In each of the five iterations, one fold was held out as the validation set, while the other four folds were used for model training. The performance metrics were then calculated on the validation set. The final cross-validation performance of a model was determined by averaging the metrics obtained from all five folds.

### Model interpretation

To ensure transparency and interpretability of the final LightGBM model, we employed the SHAP framework. SHAP is a game-theoretic approach that explains the output of any machine learning model by computing the contribution of each feature to an individual prediction. We utilized a SHAP summary plot to visualize both the global feature importance, ranked by the mean absolute SHAP value across all patients, and the directionality of each feature's impact on predicting AF recurrence.

### Statistical analysis

For normally distributed continuous variables, data are presented as mean ± standard deviation with between-group comparisons using Student's *t*-test. Continuous variables exhibiting non-normal distributions were reported as medians with interquartile ranges (IQR) and analyzed via Mann–Whitney *U* test. Category data were presented as frequencies (proportions) with between-group comparisons performed using chi-square testing. The ML algorithms were developed using Python 3.7 programming language. The LASSO algorithm and correlation analysis were conducted using the “One-key AI” platform (http://www.medai.icu/), which employed the “scipy”, “numpy”, and “sklearn” packages in Python (version 3.7). The analysis code used in this study is accessible at: https://gitee.com/wangqingbaidu/OnekeyCompo. The AUC quantified model prediction efficacy, while DeLong's test determined statistical significance of inter-model performance differences. Statistical significance was defined as a two-tailed *p*-value < 0.05.

## Results

### Patient characteristics

This study included 430 treatment-naïve patients undergoing initial radiofrequency ablation with complete medical records. Among them, 101 experienced AF recurrence within 1 year, leading to stratification into two groups based on recurrence status. Significant differences between the groups were found for: Smoking, Heart failure, CKD, NPAF, BNP, Creatinine, eGFR, Albumin, FBG, RDW-CV, Neutrophils, Lymphocyte, LAD, LVEDD, LVEF, APPLE score, SII, NLR and PLR. Table 1 presents the baseline characteristics of the included patients.

**Table 1:** Baseline characteristics.

Characteristics	Non-recurrence (*n* = 329)	Recurrence (*n* = 101)	*p* value
Age, years	63.82 ± 9.33	65.60 ± 8.20	0.125
Female, *n* (%)	149 (45.30)	37 (36.60)	0.125
BMI, kg/m^2^	25.82 ± 3.48	25.70 ± 3.63	0.878
Smoking, *n* (%)	35 (10.60)	24 (23.80)	0.001
Drinking, *n* (%)	16 (4.90)	8 (7.90)	0.242
Heart failure, *n* (%)	39 (11.90)	29 (28.70)	<0.001
Hypertension, *n* (%)	183 (55.60)	51 (50.50)	0.365
DM, *n* (%)	45 (13.70)	20 (19.80)	0.133
CHD, *n* (%)	99 (30.10)	37 (36.60)	0.216
Stroke, *n* (%)	46 (14.0)	16 (15.80)	0.642
Sleep disorders, *n* (%)	7 (2.10)	2 (2.00)	0.928
CKD, *n* (%)	5 (1.5)	10 (9.9)	<0.001
NPAF, *n* (%)	116 (35.30)	62 (61.40)	<0.001
BNP, pg/ml	87 (41.00, 191.50)	179 (98.65, 370.50)	<0.001
UA, (µmol/L)	327 (270, 389.50)	332 (267.50, 411)	0.303
Creatinine, (µmol/L)	63.00 (53.00, 74.00)	67.00 (59.00, 79.00)	0.015
eGFR (ml/min/1.73 m^2^)	111.77 (92.82, 137.10)	103.80 (85.31, 122.81)	0.011
TG, mmol/L	1.28 (0.96, 1.85)	1.23 (0.91, 1.64)	0.358
LDL-C, mmol/L	2.63 (2.02, 3.22)	2.44 (1.74, 3.12)	0.066
HDL-C, mmol/L	1.17 (1.01, 1.40)	1.14 (0.99, 1.40)	0.796
GGT, U/L	24.00 (18.00, 34.00)	25.00 (17.00, 38.50)	0.553
Albumin, g/L	42.10 (39.70, 45.20)	41.10 (38.90, 43.60)	0.012
FBG (mmol/L)	5.30 (4.80, 6.00)	5.50 (4.80, 7.60)	0.017
WBC count (×10^9^/L)	5.91 (5.11, 6.94)	5.68 (4.81, 6.89)	0.316
RBC count (×10^9^/L)	4.54 (4.23, 4.91)	4.49 (4.18, 4.92)	0.403
HGB, g/L	140.64 ± 15.54	138.68 ± 18.46	0.607
RDW-CV,%	12.50 (12.10, 12.80)	12.70 (12.40, 13.10)	0.002
Neutrophils (×10^9^/L)	3.52 (2.91, 4.35)	3.77 (3.08, 4.85)	0.039
Lymphocyte (×10^9^/L)	1.79 (1.45, 2.21)	1.42 (1.13, 1.88)	<0.001
Monocytes (×10^9^/L)	0.37 (0.30, 0.46)	0.38 (0.29, 0.46)	0.843
Platelet count (×10^9^/L)	212.00 (180.50, 254.00)	202.00 (177.50, 245.00)	0.146
FT3, pmol/L	5.06 (4.66, 5.62)	4.99 (4.53, 5.43)	0.093
FT4, pmol/L	17.65 (15.39, 19.46)	18.06 (15.57, 19.87)	0.193
TSH, μIU/ml	1.68 (1.15, 2.53)	1.59 (1.05, 3.25)	0.847
LAD, mm	33.80 (30.60, 38.00)	38.45 (33.48, 42.00)	<0.001
LVEDD, mm	49.00 (46.50, 51.60)	50.00 (47.00, 53.50)	0.015
LVEF, %	64.00 (60.00, 68.00)	62.00 (57.50, 66.00)	<0.001
CHA_2_DS_2_-VASc score	2.50 ± 1.55	2.44 ± 1.48	0.722
APPLE score	1.18 ± 0.99	1.86 ± 1.36	<0.001
SII	402.55 (293.13, 579.98)	581.13 (396.61, 741.88)	<0.001
NLR	1.91 (1.45, 2.66)	2.79 (1.91, 3.68)	<0.001
PLR	118.30 (93.70, 150.31)	138.43 (122.06, 173.08)	<0.001
MHR	8.07 (6.13, 10.80)	8.53 (6.01, 11.47)	0.910

BMI, body mass index; DM, diabetes mellitus; CHD, coronary heart disease; CKD, chronic kidney disease; NPAF, nonparoxysmal atrial fibrillation; BNP, B-type natriuretic peptide; UA, uric acid; eGFR, estimated glomerular filtration rate; TG, total glyceride; LDL, low density lipoprotein cholesterol; HDL, high-density lipoprotein cholesterol; GGT, gamma-glutamyl transferase; FBG, fasting blood glucose; RDW-CV, red cell distribution width-coefficient of variation; FT_3_, free triiodothyronine; FT_4_, free tetraiodothyronine; TSH, thyroid-stimulating hormone; LAD, left atrial diameter; LVEDD, left ventricular end-diastolic diameter; LVEF, left ventricular ejection fraction; SII, systemic immune inflammation; NLR, neutrophil-lymphocyte ratio; PLR, platelet-lymphocyte ratio; MHR, monocyte-to- high-density lipoprotein ratio.

### Feature selection and model building

**Figure 2:**
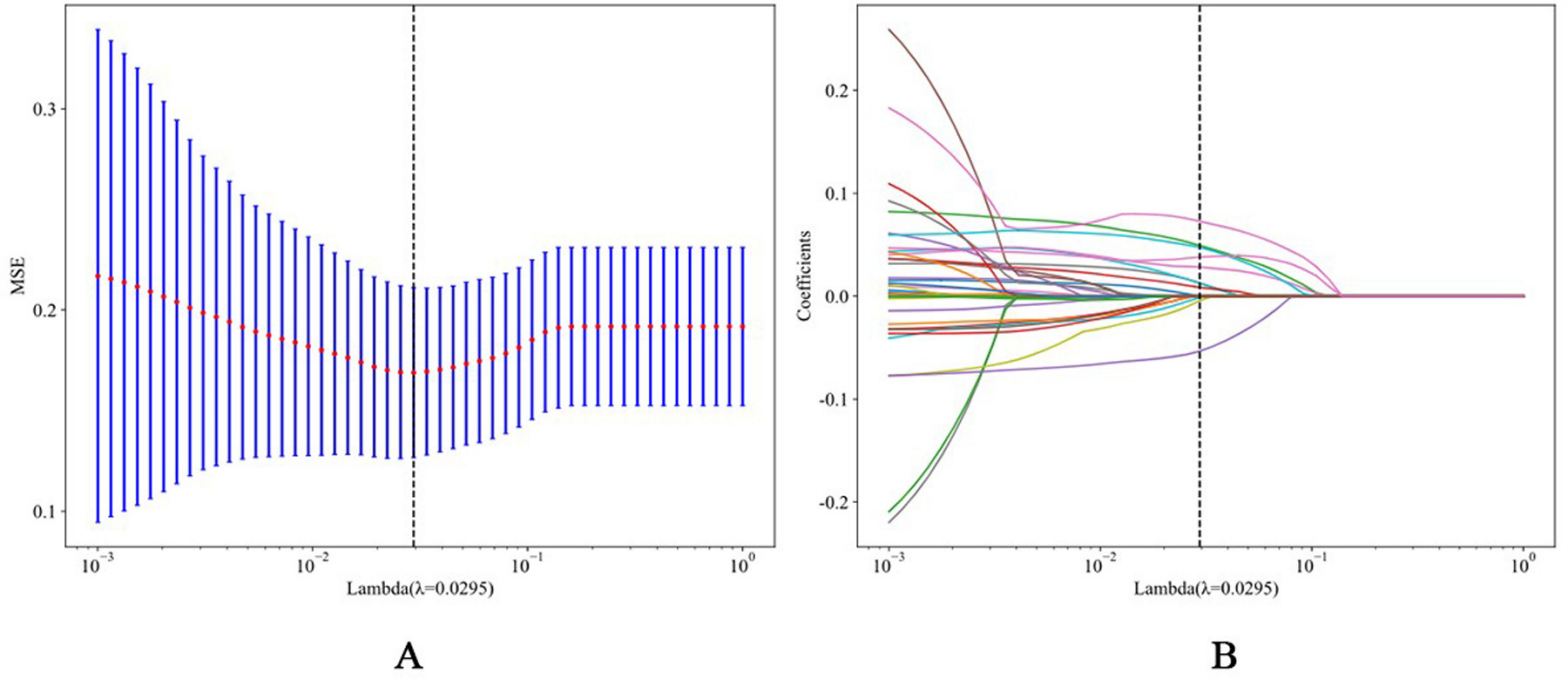
**(A)** The process of feature selection. We used the least absolute shrinkage and selection operator (LASSO) regression model with penalty parameter tuning conducted by fivefold cross validation according to minimum criteria. Selection of the tuning parameter (*λ*). Based on the minimum criteria, the vertical dotted line is plotted at the optimal value *λ* = 0.0110. (B) The vertical line was plotted with 10 selected features.

Through Spearman correlation analysis and LASSO regression with stratified fivefold cross-validation (Figures 2A,B), we identified 10 potential predictors of AF recurrence post-RFCA from the initial 43 variables (Table 2). These predictors were subsequently used to construct the final model.

**Table 2:** Feature selection results and coefficients for each feature.

Variable name	Coefficient
NLR	0.079150
NPAF	0.039257
APPLE score	0.036335
ALB	−0.033608
FBG	0.028001
LAD	0.013889
BNP	0.012901
Lymphocyte	−0.011794
Smoking	0.011365
CKD	0.004392

NLR, neutrophil-lymphocyte ratio; NPAF, nonparoxysmal atrial fibrillation; ALB, albumin; FBG, fasting blood glucose; LAD, left atrial diameter; BNP, B-type natriuretic peptide; CKD, chronic kidney disease.

The dataset was divided into a training set (70%) and a testing set (30%) using a stratified random split based on the AF recurrence. To confirm the validity of this split, we compared the baseline characteristics between the two sets. As shown in Supplementary Table S1, there were no significant statistical differences for the vast majority of variables, indicating that the training and testing sets were well-balanced and comparable. This provides astrong basis for assessing the model's generalizability.

Four machine learning algorithms were employed to develop prediction models in the training set. To ensure optimal performance and prevent data leakage, hyperparameters for each algorithm were tuned using a grid search with stratified 5-fold cross-validation. Stratification was based on the AF recurrence outcome to ensure that the proportion of positive and negative cases was maintained across all folds, a crucial step for handling potential class imbalance. For each model, we defined a grid of relevant hyperparameters and sought the combination that maximized the mean AUC across the validation folds. The final optimized hyperparameters for all models are detailed in Supplementary Table S2.

To compare the performance and stability of the candidate models during the training phase, we visualized the distribution of AUCs from the stratified cross-validation process (Supplementary Figure S1). The boxplot clearly demonstrates that the LightGBM model achieved not only the highest median AUC but also showed a relatively tight interquartile range, suggesting its superior predictive power and robustness compared to the other models.

The finalized models, using their optimized hyperparameters, were then evaluated on the independent testing set. To visualize the generalization capability of each model, we compared their performance on the training and testing sets (Supplementary Figure S2). The plot shows that the LightGBM and SVM models maintained a smaller performance gap compared to other models, providing visual confirmation of their superior stability and lower risk of overfitting.

**Figure 3:**
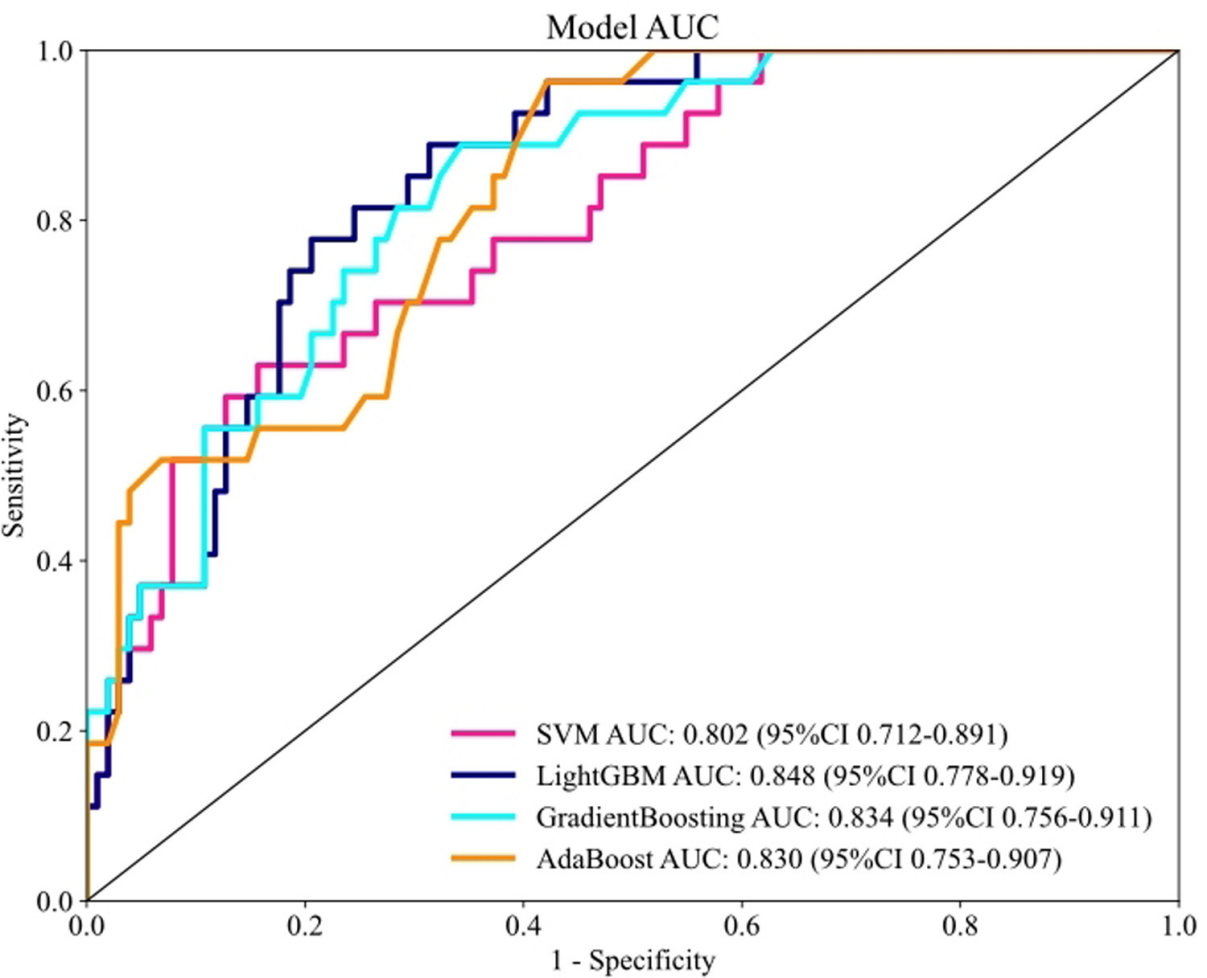
Performance for machine learning models in the testing set based on the AUC of the ROC curve.

The receiver operating characteristic (ROC) curves and corresponding AUC values for each model in the testing set are presented in Figure 3. The results demonstrated that the LightGBM model achieved a numerically high AUC of 0.848 compared to the other models: GradientBoosting (AUC = 0.834), AdaBoost (AUC = 0.830), and SVM (AUC = 0.802). Table 3 summarizes the predictive performance across training and testing datasets. Although a DeLong test indicated that the AUC of the LightGBM model was not statistically significantly different from that of the Gradient Boosting model (*p* = 0.21), we selected the LightGBM model for further analysis given its combination of being the top numerical performer and known computational efficiency.

**Table 3:** Comparison of the performance of machine learning models in the training and testing set.

Set	Model	Accuracy	AUC (95% CI)	Sensitivity	Specificity
Training set	SVM	0.831	0.882 (0.835, 0.930)	0.838	0.828
	LightGBM	0.824	0.919 (0.885, 0.952)	0.919	0.793
	GradientBoosting	0.887	0.923 (0.886, 0.960)	0.784	0.921
	AdaBoost	0.761	0.850 (0.803, 0.898)	0.851	0.731
Testing set	SVM	0.791	0.802 (0.712, 0.891)	0.593	0.843
	LightGBM	0.721	0.848 (0.778, 0.919)	0.852	0.686
	GradientBoosting	0.713	0.834 (0.756, 0.911)	0.852	0.676
	AdaBoost	0.667	0.830 (0.753, 0.907)	0.889	0.608

SVM, support vector machine; KNN, K-nearest neighbor; RF, random forest; LightGBM, light gradient boosting machine; AdaBoost, adaptive boosting.

### Interpretation of the optimal model with SHAP

**Figure 4:**
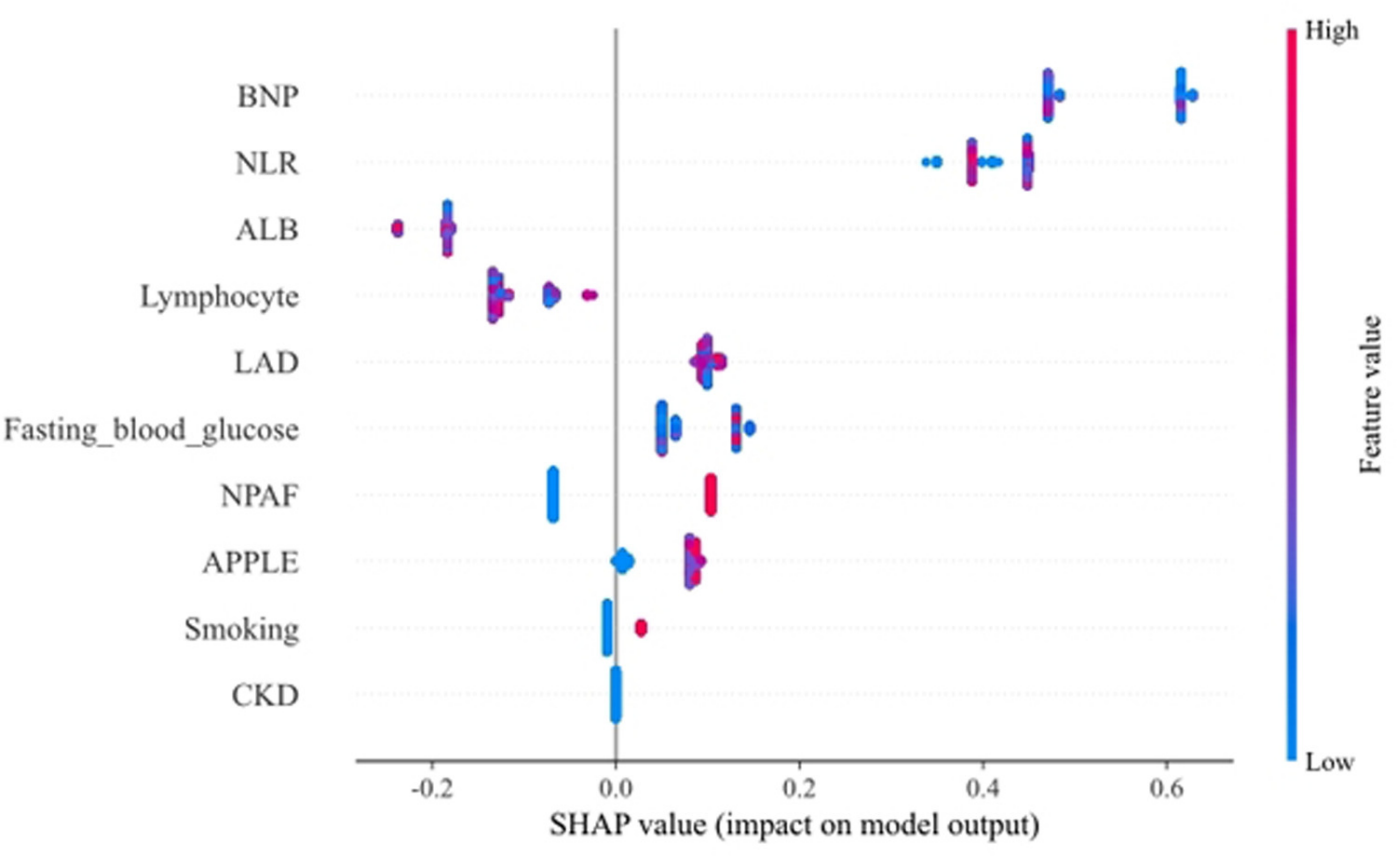
SHAP summary plot for the LightGBM model. Each point on the plot represents a single patient from the testing set. The *y*-axis lists the features, ordered by their global importance (mean absolute SHAP value). The *x*-axis represents the SHAP value, indicating the feature's impact on the model output (a positive value increases the prediction of recurrence). The color of each point corresponds to the feature's value for that patient, from low (blue) to high (red). NLR, neutrophil-lymphocyte ratio; NPAF, nonparoxysmal atrial fibrillation; LAD, left atrial diameter; BNP, B-type natriuretic peptide; CKD, chronic kidney disease; ALB, albumin.

**Figure 5:**
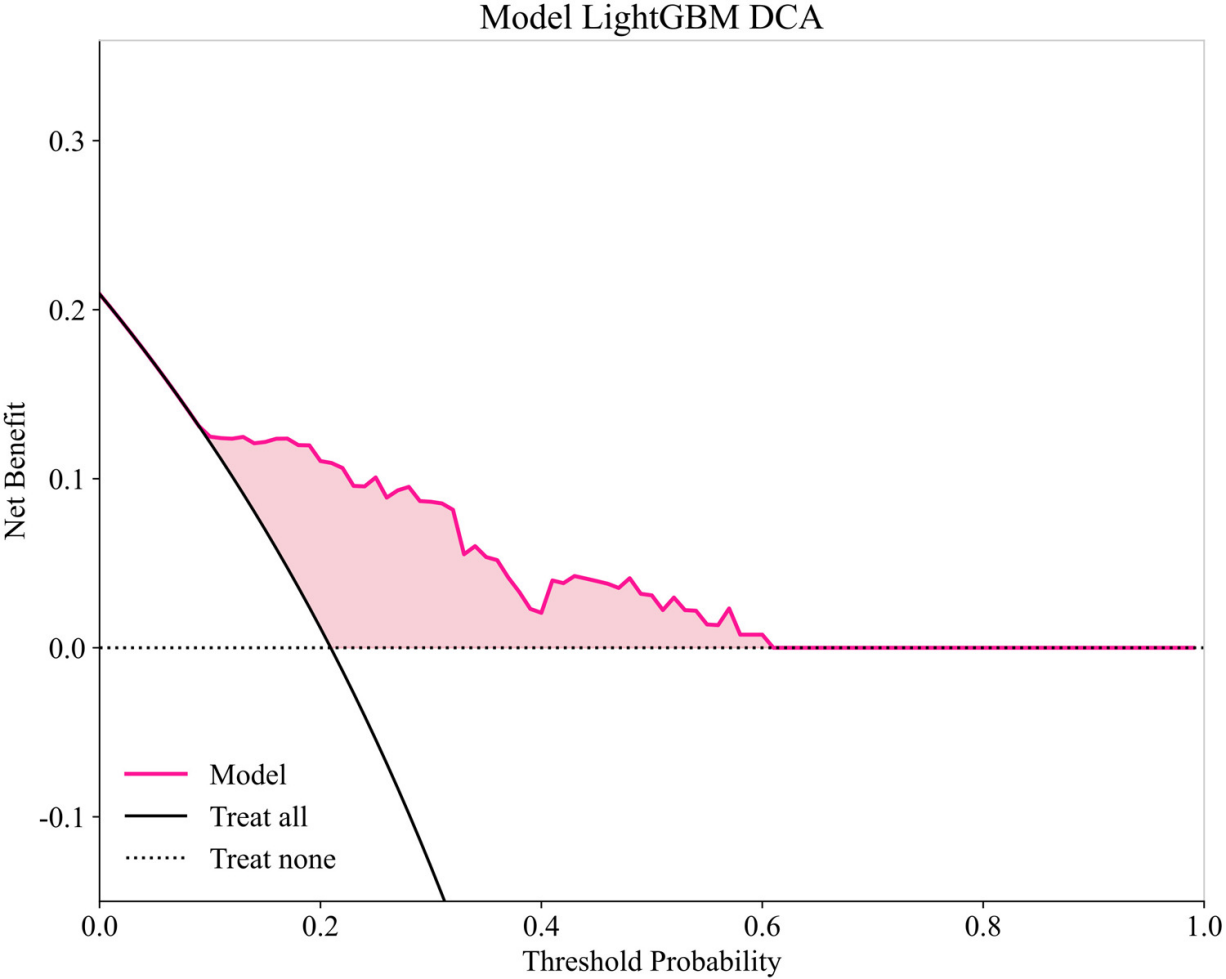
Decision curve analysis of LightGBM model in testing set.

To provide a detailed and clinically meaningful interpretation of the best-performing LightGBM model, we conducted a SHAP analysis on the testing set. The SHAP summary plot (Figure 4) illustrates the global importance and impact of each of the 10 selected predictors on the model's output. The analysis identified BNP as the most influential predictor. As shown in Figure 4, high BNP levels (represented by red dots) consistently yielded high positive SHAP values, indicating a strong association with an increased risk of AF recurrence. The NLR was the second most important feature, where high values clearly drove the prediction towards recurrence. Interestingly, the SHAP analysis also highlighted significant protective factors. Higher levels of ALB and lymphocyte count were associated with negative SHAP values (pushing the prediction towards no recurrence), suggesting they reduce the likelihood of postoperative AF recurrence. This finding is clinically coherent, as a low lymphocyte count is a key component of an elevated, high-risk NLR. Other predictors aligned with established clinical knowledge. A larger LAD, higher FBG, the presence of NPAF, a higher APPLE score, and a history of smoking were all identified as risk factors that increase the model's predicted probability of recurrence. This granular interpretation afforded by SHAP enhances the model's transparency and potential for clinical decision support. Figure 5 displays the DCA results of the LightGBM model in the test set, demonstrating clinical utility across the 0.05–0.60 threshold probability range.

## Discussion

As a well-established rhythm management strategy, catheter ablation provides safe treatment for symptomatic AF patients (11). The effectiveness of atrial fibrillation catheter ablation persists as a clinical challenge, demonstrating 20%–45% recurrence rates after initial procedures (12). Precise AF recurrence prediction optimizes clinical decision-making and patient selection for ablation. Consequently, preprocedural quantification of personalized AF recurrence risk is imperative for ablation candidates. Here, we developed and compared several machine learning models for predicting late recurrence following RFCA. Our findings suggest that ML models, particularly LightGBM, demonstrate modest predictive performance and warrant further investigation.

Our study's findings should be viewed within the rapidly advancing landscape of artificial intelligence in clinical electrophysiology. In recent years, AI has demonstrated remarkable capabilities across this field. For instance, deep learning models, such as convolutional and recurrent neural networks, have achieved expert-level performance in detecting and classifying arrhythmias from 12-lead ECGs or wearable device recordings (13). Other significant efforts have focused on predicting the long-term success of catheter ablation for atrial fibrillation by integrating clinical, electrophysiological, and imaging data (14). Furthermore, AI is increasingly used for personalized risk stratification, identifying patients at high risk for events like sudden cardiac death or thromboembolism, thereby guiding preventive strategies (15).

Machine learning is rapidly advancing with expanding applications in cardiovascular medicine (16). These analytical methods autonomously identify clinically significant patterns through iterative data learning, eliminating the need for predefined search parameters. ML's key advantage lies in autonomously modeling complex nonlinear interactions across patient characteristics and comorbidities, eliminating predefined variable constraints required in traditional methods like logistic regression. Liu et al. (8) developed a deep learning-based model (AUC = 0.82) using CT imaging to detect extra-pulmonary vein AF triggers for predicting post-ablation arrhythmia recurrence. Shade et al. (17) developed a deep learning model using late gadolinium-enhanced CMR (LGE-CMR) imaging to preprocedurally predict AF recurrence risk (AUC = 0.82). Their methodology extracted high-risk features from simulated LGE-CMR data. Although their machine learning model achieved satisfactory predictive accuracy, its dependency on sophisticated and high-cost diagnostics could hinder broad clinical adoption. Therefore, developing efficient and generalizable prediction models using machine learning with readily available and low-cost indicators is crucial.

Inflammation significantly contributes to atrial fibrillation by promoting both electrical and structural atrial remodeling (18). Recent studies have identified novel inflammatory markers (e.g., NLR, SII, PLR) that are significantly associated with atrial fibrillation recurrence. Moreover, these novel inflammatory markers can be derived from routine clinical tests. However, few existing prediction models have incorporated these emerging inflammatory indicators for AF recurrence prediction. To our knowledge, this is the first study to integrate novel inflammatory biomarkers into an ML model for AF recurrence prediction. In this study, we compared the performance of four machine learning algorithms in predicting atrial fibrillation recurrence after RFCA. Recognizing the methodological constraints of retrospective single-center designs, we employed: (a) protocolized data quality control measures to limit information bias, and (b) multivariable regression modeling to account for confounding variables, thereby strengthening inference validity and reducing institution-specific bias effects. To safeguard against model overfitting, we deployed these methodological safeguards: Firstly, we employed LASSO regression for feature selection, utilizing its L1 regularization properties to reduce model complexity and suppress overfitting. Secondly, we implemented stratified 5-fold cross-validation with repeated random partitioning to quantify generalization performance during model training, thereby mitigating overfitting risks through robust out-of-sample validation. Finally, we developed predictive models using four distinct machine learning algorithms: support vector machine (SVM), Gradient Boosting, LightGBM, and AdaBoost. Through comparative performance assessment across multiple algorithms, we selected the optimal model based on prespecified metrics, thereby enhancing external validity.

Our findings indicate that several machine learning models, particularly those based on gradient boosting, can achieve modest predictive performance. While the LightGBM model yielded the numerically highest AUC (0.848), it is important to emphasize that its performance was not statistically superior to other competitive models like Gradient Boosting, as evidenced by the DeLong test. The overlapping 95% confidence intervals for these models further support this observation. Therefore, the data suggest that multiple models perform comparably. Our choice of LightGBM for deeper analysis is based on its combination of being the top numerical performer and its well-established advantages in computational efficiency, which are practical considerations for potential future applications.

Interpretation of the LightGBM model using SHAP values revealed the relative importance and directional impact of the 10 predictors (Figure 4). The analysis identified BNP, NLR, ALB, and lymphocyte count as the four most influential factors. The NLR serves as an established biomarker of systemic inflammatory burden and oxidative stress status. Guo et al. (19) demonstrated that elevated postprocedural NLR independently predicts recurrent lone atrial fibrillation. Bazoukis et al. (20) reported significantly elevated post-ablation NLR in patients experiencing late atrial fibrillation recurrence, with NLR > 3.9 predicting recurrence at 70% sensitivity and 38% specificity. Moreover, a systematic meta-analysis by Shao et al. (21) confirmed that elevated NLR—whether measured pre-procedurally or post-intervention—consistently correlates with heightened risks of both incident and recurrent atrial fibrillation. NLR reflects systemic stress and inflammatory status, demonstrating the strongest predictive contribution in our study. The SHAP analysis confirmed that a higher lymphocyte count was significantly associated with a reduced risk of AF recurrence, indicating its protective effect (Figure 4). A prospective cohort study (22) further demonstrated that elevated absolute counts of WBC, neutrophils, and monocytes each independently associated with increased atrial fibrillation risk, whereas higher lymphocyte counts exhibited an inverse association. In addition, histopathological analyses of atrial tissue biopsies from AF patients reveal prominent lymphocytic and mononuclear cell infiltration—a distinct inflammatory substrate absent in sinus rhythm controls, as consistently documented in prior studies (23, 24). Consequently, patients exhibiting elevated NLR or lymphocytopenia merit heightened clinical vigilance and targeted risk stratification.

Established risk factors for arrhythmia recurrence include persistent AF and left atrial enlargement (25). Previous reports have demonstrated that LAD is a predictor of recurrences after RFCA (26). Left atrial enlargement contributes to structural and electrical remodeling within the atrium, thereby promoting the persistence and perpetuation of atrial arrhythmias (27). The risk of arrhythmia recurrence was greater in patients with persistent AF compared to those with paroxysmal AF (28). Persistent AF contributes to atrial fibrosis, promoting both structural and electrical remodeling, which ultimately perpetuates the arrhythmia (29).

Albumin, a key plasma protein, modulates inflammation, sustains colloidal osmotic pressure, and facilitates the transport of diverse endogenous and exogenous compounds (30). Our SHAP analysis prominently identified serum albumin level as the third most important predictor and a strong independent protective factor (Figure 4), where higher levels were robustly associated with a lower risk of recurrence. Furthermore, albumin reflects nutritional status, a parameter independently associated with atrial fibrillation recurrence (31). Consequently, patients identified as high-risk who present with hypoalbuminemia warrant intensified clinical monitoring and targeted interventions.

The APPLE score, proposed by Kornej et al. (10), is a risk stratification tool (range 0–5) for predicting AF recurrence after catheter ablation, assigning 1 point per risk factor. Independent external validation studies confirm the robust predictive performance of the APPLE score for AF recurrence following RFCA and demonstrate its ability to effectively stratify patients into distinct low-, moderate-, and high-risk categories for recurrence (32, 33).

BNP, a hormone primarily secreted by ventricular cardiomyocytes in response to wall stress, exerts diuretic and natriuretic effects. Elevated circulating BNP levels are a recognized feature in patients with AF. Our model identified preprocedural BNP as the single most powerful predictor of AF recurrence (Figure 4). This aligns with and reinforces the findings of numerous investigations that have established the robust predictive utility of BNP for post-ablation outcomes (34, 35).

The cardiotoxic effects of smoking are multifactorial, stemming from over 4,000 constituents in cigarette smoke, with nicotine and carbon monoxide being the primary mediators of direct pathophysiological injury. Nicotine elevates sympathetic tone and promotes atrial fibrosis, the latter involving induction of collagen III mRNA expression (36). Collectively, these pro-fibrotic and neurohormonal effects culminate in atrial conduction abnormalities and enhanced automaticity (37). Conversely, smoking may promote tissue inflammation, evidenced by elevated blood levels of Interleukin-6 (IL-6), Tumor Necrosis Factor-alpha (TNF-α), and C-reactive protein (CRP) (38). This inflammatory state can subsequently contribute to fibrosis and increase susceptibility to arrhythmias (39). A recent study demonstrated that smoking is associated with increased AF recurrence risk after PVI, regardless of AF type (paroxysmal or persistent) (40). Therefore, smoking cessation should be encouraged in all AF patients, particularly those undergoing ablation, to achieve long-term benefits for sinus rhythm maintenance and overall health.

Impaired renal function inevitably leads to systemic toxin accumulation and tissue damage, adversely affecting cardiac remodeling. Previous studies indicate that chronic kidney disease (CKD) is a risk factor not only for new-onset atrial fibrillation (AF) (41), but also for AF recurrence following catheter ablation (42). Therefore, particular attention should be given to AF patients with CKD.

Abnormally elevated fasting glucose often indicates diabetes or prediabetes (impaired fasting glucose). Prior research has established that dysglycemia detrimentally alters the biatrial electrophysiological substrate, manifesting as prolonged intra-atrial conduction, diminished bipolar voltage, and elevated recurrence risk following catheter ablation (43). Therefore, aggressive glycemic control is warranted in AF patients with impaired fasting glucose or diabetes to maintain long-term sinus rhythm.

Several limitations of this study warrant consideration. First, our model was developed using data from a single center, which inherently limits the sample size and may introduce selection bias, potentially restricting the model's external validity. To confirm its generalizability and clinical utility, future validation in larger, independent, and multicenter cohorts is essential. Second, the ascertainment of AF recurrence relied on scheduled monitoring and patient-reported symptoms rather than continuous surveillance with implantable loop recorders. This approach may have missed asymptomatic episodes, leading to a potential underestimation of the true recurrence rate. Third, the retrospective design of our study precluded the detailed collection of pre-procedural medication data, such as the specifics of antiarrhythmic drug therapy or the use of beta-blockers and renin-angiotensin system inhibitors. The absence of this information represents a potential unmeasured confounding factor. Future prospective studies are needed to meticulously collect and integrate these variables into predictive models to refine risk stratification. Fourth, we excluded patients with primary cardiomyopathy, a group with distinct pathophysiology and higher ablation failure rates. While this exclusion enhances the model's precision for the intended target population (i.e., AF without primary cardiomyopathy), it concurrently limits its applicability to this specific high-risk subgroup. Therefore, the development of dedicated prediction models for patients with underlying cardiomyopathies is warranted. Finally, our selected LightGBM model exhibits a crucial performance trade-off, prioritizing high sensitivity (0.852) at the expense of modest specificity (0.686). This performance profile results in a higher false-positive rate, which could lead to unnecessary downstream testing or increased patient anxiety. Consequently, the model is best positioned as a screening tool to identify patients warranting closer surveillance, rather than as a standalone diagnostic instrument. Final treatment decisions must continue to integrate the model's output with comprehensive clinical judgment. Future research should focus on improving specificity, perhaps by incorporating novel biomarkers or longitudinal data, to enhance the model's overall clinical utility.

## Conclusion

In this single-center study, we developed and compared several machine learning models for predicting late AF recurrence post-RFCA. The models demonstrated modest predictive performance, with no single algorithm proving statistically superior to others. The LightGBM model, while being the top numerical performer, primarily showed potential due to its computational efficiency. Crucially, these findings require rigorous external and prospective validation to assess their generalizability and true clinical utility. At present, such models should be considered exploratory tools that may help generate hypotheses for future studies rather than being used for direct clinical decision-making.

## Data Availability

The original contributions presented in the study are included in the article/Supplementary Material, further inquiries can be directed to the corresponding author.
