# Supplementary material for: Machine learning-based prediction model for recurrence after radiofrequency catheter ablation in patients with atrial fibrillation
Source: Front Cardiovasc Med. 2025 Aug 8;12:1642409. doi: 10.3389/fcvm.2025.1642409 (PMC12370652; doi:10.3389/fcvm.2025.1642409)
Supplement: Supplementary file 1 [file Datasheet1.docx]

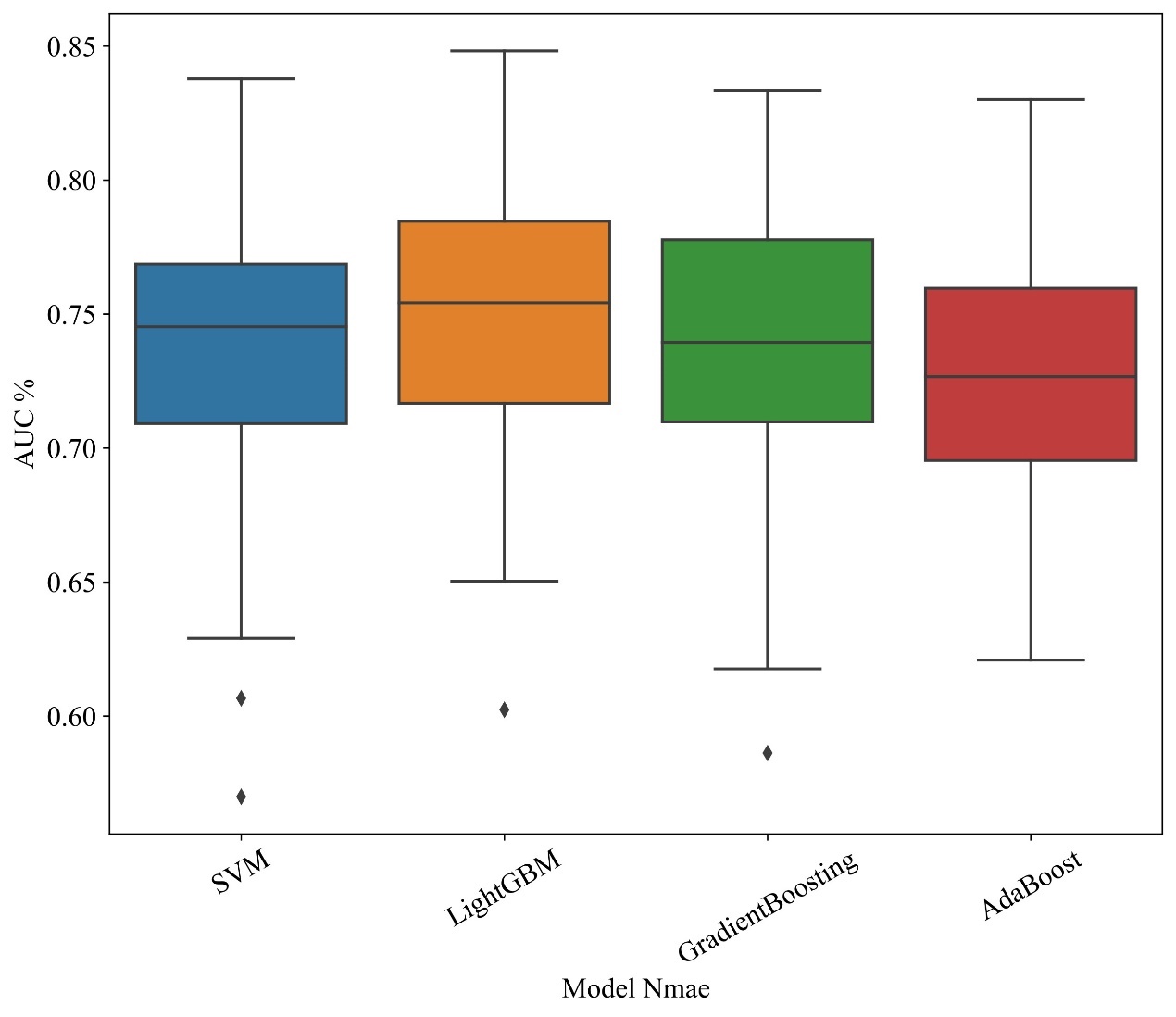


**Supplementary Figure S1.** Performance of machine learning models in cross-validation.

Boxplot of Area Under the Curve (AUC) values from stratified 5-fold cross-validation on the training set for four models. The LightGBM model achieved the highest median AUC, indicating superior performance.


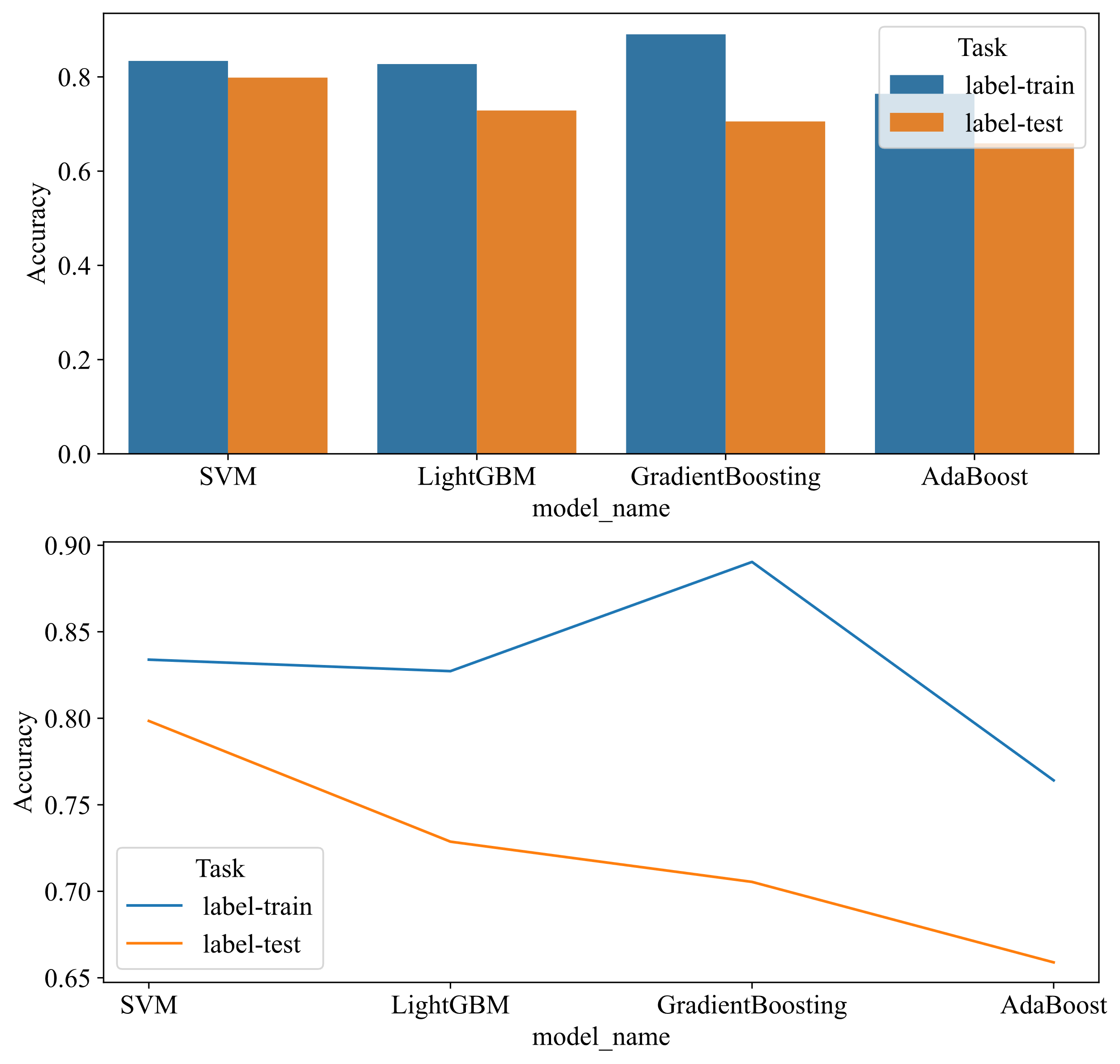


**Supplementary Figure S2.** Generalization performance of the four candidate models.

Figure Legend: This line plot compares the final accuracy of the Support Vector Machine (SVM), LightGBM, GradientBoosting, and AdaBoost models on the training set (blue line) and the independent testing set (orange line). The vertical gap between the two lines for each model represents its generalization gap. Smaller gaps indicate better model stability and less overfitting. The plot visually confirms the robust generalization of the LightGBM model.

**Supplementary Table S1.** Baseline characteristics of patients in the training and testing sets.

| Feature name | All | Test | Train | P value |
| --- | --- | --- | --- | --- |
| Age | 64.240±9.099 | 64.481±8.936 | 64.136±9.181 | 0.628 |
| BMI | 25.792±3.516 | 25.572±3.445 | 25.886±3.547 | 0.414 |
| LAD | 39.060±5.957 | 38.622±5.636 | 39.248±6.089 | 0.471 |
| LVEDD | 49.408±4.775 | 48.818±4.268 | 49.660±4.962 | 0.218 |
| LVEF | 62.416±8.337 | 61.977±8.758 | 62.605±8.157 | 0.335 |
| BNP | 184.801±223.608 | 173.678±198.200 | 189.568±233.803 | 0.87 |
| WBC | 6.096±1.535 | 5.943±1.537 | 6.162±1.533 | 0.236 |
| RBC | 4.561±0.521 | 4.532±0.480 | 4.574±0.538 | 0.412 |
| HGB | 140.181±16.272 | 139.519±15.160 | 140.465±16.742 | 0.332 |
| RDW_CV | 13.143±1.857 | 12.963±0.732 | 13.220±2.164 | 0.201 |
| PLT | 217.326±57.942 | 217.659±54.582 | 217.183±59.411 | 0.738 |
| SII | 511.622±282.130 | 511.865±303.100 | 511.518±273.178 | 0.468 |
| Lymphocyte | 1.782±0.593 | 1.759±0.582 | 1.792±0.598 | 0.672 |
| MC | 0.388±0.126 | 0.375±0.133 | 0.394±0.123 | 0.059 |
| NC | 3.802±1.298 | 3.707±1.358 | 3.842±1.271 | 0.113 |
| NLR | 2.374±1.192 | 2.351±1.211 | 2.383±1.185 | 0.42 |
| PLR | 132.733±48.963 | 135.410±52.857 | 131.586±47.242 | 0.693 |
| MHR | 8.940±4.080 | 8.433±4.044 | 9.157±4.083 | 0.039 |
| FBG | 5.935±2.205 | 6.229±2.258 | 5.809±2.173 | 0.1 |
| UA | 332.609±90.039 | 325.566±83.406 | 335.628±92.706 | 0.478 |

**Supplementary Table S1. Baseline characteristics of patients in the training and testing sets.**

| Feature name | All | Test | Train | P value |
| --- | --- | --- | --- | --- |
| Ser | 65.667±16.903 | 64.171±14.235 | 66.309±17.910 | 0.474 |
| eGFR | 114.767±33.695 | 116.115±31.877 | 114.189±34.481 | 0.523 |
| TG | 1.516±0.942 | 1.426±0.816 | 1.555±0.990 | 0.073 |
| GGT | 33.793±33.060 | 29.876±23.998 | 35.472±36.164 | 0.065 |
| ALB | 42.038±3.996 | 41.612±3.874 | 42.220±4.039 | 0.336 |
| CHA_2_DS_2_-VASc score | 2.488±1.528 | 2.504±1.606 | 2.482±1.496 | 0.973 |
| APPLE | 1.342±1.119 | 1.318±1.097 | 1.352±1.129 | 0.768 |
| Gender |  |  |  | 0.718 |
| 0 | 244(56.744) | 71(55.039) | 173(57.475) |  |
| 1 | 186(43.256) | 58(44.961) | 128(42.525) |  |
| NPAF |  |  |  | 0.381 |
| 0 | 252(58.605) | 71(55.039) | 181(60.133) |  |
| 1 | 178(41.395) | 58(44.961) | 120(39.867) |  |
| Hypertension |  |  |  | 0.434 |
| 0 | 196(45.581) | 63(48.837) | 133(44.186) |  |
| 1 | 234(54.419) | 66(51.163) | 168(55.814) |  |
| CHR |  |  |  | 0.946 |
| 0 | 294(68.372) | 89(68.992) | 205(68.106) |  |
| 1 | 136(31.628) | 40(31.008) | 96(31.894) |  |
| HF |  |  |  | 0.261 |
| 0 | 362(84.186) | 113(87.597) | 249(82.724) |  |
| 1 | 68(15.814) | 16(12.403) | 52(17.276) |  |
| Stroke |  |  |  | 0.976 |
| 0 | 368(85.581) | 111(86.047) | 257(85.382) |  |
| 1 | 62(14.419) | 18(13.953) | 44(14.618) |  |

**Supplementary Table S1. Baseline characteristics of patients in the training and testing sets.**

| Feature name | All | Test | Train | P value |
| --- | --- | --- | --- | --- |
| CKD |  |  |  | 0.378 |
| 0 | 421(97.907) | 128(99.225) | 293(97.342) |  |
| 1 | 9(2.093) | 1(0.775) | 8(2.658) |  |
| DM |  |  |  | 0.142 |
| 0 | 365(84.884) | 104(80.620) | 261(86.711) |  |
| 1 | 65(15.116) | 25(19.380) | 40(13.289) |  |
| Smoking |  |  |  | 0.199 |
| 0 | 371(86.279) | 116(89.922) | 255(84.718) |  |
| 1 | 59(13.721) | 13(10.078) | 46(15.282) |  |
| Drinking |  |  |  | 0.436 |
| 0 | 406(94.419) | 124(96.124) | 282(93.688) |  |
| 1 | 24(5.581) | 5(3.876) | 19(6.312) |  |
| SD |  |  |  | 1.0 |
| 0 | 421(97.907) | 126(97.674) | 295(98.007) |  |
| 1 | 9(2.093) | 3(2.326) | 6(1.993) |  |

BMI: Body mass index; DM: Diabetes mellitus; CHD: Coronary heart disease; **CKD: Chronic kidney disease;** NPAF: Nonparoxysmal atrial fibrillation; BNP: B-type natriuretic peptide; UA: Uric acid; **eGFR: estimated glomerular filtration rate;** TG: Total glyceride; LDL: Low density lipoprotein cholesterol; HDL: High-density lipoprotein cholesterol; GGT: Gamma-Glutamyl Transferase; FBG: Fasting blood glucose; RDW-CV: Red cell distribution width-Coefficient of variation; FT_3_: Free triiodothyronine; FT_4_: Free tetraiodothyronine; TSH: Thyroid-stimulating hormone; LAD: Left atrial diameter; LVEDD: Left ventricular end-diastolic diameter; LVEF: Left ventricular ejection fraction; SII: Systemic immune inflammation; NLR: Neutrophil-lymphocyte ratio; PLR: Platelet-lymphocyte ratio; **MHR: Monocyte-to- high-density lipoprotein ratio**

new_models['NaiveBayes'] = GaussianNB()

new_models['SVM'] = SVC(class_weight = 'balanced', probability=True, random_state=0)

new_models['LightGBM'] = LGBMClassifier(class_weight = 'balanced', n_estimators=10, max_depth=3, objective='binary')

new_models['GradientBoosting'] = GradientBoostingClassifier(n_estimators=10, random_state=0, max_depth=3)

new_models['AdaBoost'] = AdaBoostClassifier(algorithm='SAMME.R', n_estimators=10, random_state=0)
